# Sildenafil attenuates hypoxic pulmonary remodelling by inhibiting bone marrow progenitor cells

**DOI:** 10.1111/jcmm.13026

**Published:** 2016-11-18

**Authors:** Shirley Favre, Elisa Gambini, Patrizia Nigro, Alessandro Scopece, Paola Bianciardi, Anna Caretti, Giulio Pompilio, Antonio F. Corno, Giuseppe Vassalli, Ludwig K. von Segesser, Michele Samaja, Giuseppina Milano

**Affiliations:** ^1^ Laboratory of Cardiovascular ResearchDepartment of Surgery and AnesthesiologyUniversity Hospital LausanneLausanneSwitzerland; ^2^ Vascular Biology and Regenerative Medicine UnitCentro Cardiologico Monzino–IRCCSMilanItaly; ^3^ Department of Health ScienceUniversity of MilanMilanItaly; ^4^ Glenfield HospitalLeicesterUK; ^5^ Laboratory of Molecular and Cellular CardiologyDepartments of Cardiology and Heart SurgeryLausanneSwitzerland

**Keywords:** chronic hypoxia, sildenafil, bone marrow progenitor cells, pulmonary hypertension, c‐kit cells, CXCR4 receptor

## Abstract

The recruitment of bone marrow (BM)‐derived progenitor cells to the lung is related to pulmonary remodelling and the pathogenesis of pulmonary hypertension (PH). Although sildenafil is a known target in PH treatment, the underlying molecular mechanism is still elusive. To test the hypothesis that the therapeutic effect of sildenafil is linked to the reduced recruitment of BM‐derived progenitor cells, we induced pulmonary remodelling in rats by two‐week exposure to chronic hypoxia (CH, 10% oxygen), a trigger of BM‐derived progenitor cells. Rats were treated with either placebo (saline) or sildenafil (1.4 mg/kg/day ip) during CH. Control rats were kept in room air (21% oxygen) with no treatment. As expected, sildenafil attenuated the CH‐induced increase in right ventricular systolic pressure and right ventricular hypertrophy. However, sildenafil suppressed the CH‐induced increase in c‐kit^+^ cells in the adventitia of pulmonary arteries. Moreover, sildenafil reduced the number of c‐kit^+^ cells that colocalize with tyrosine kinase receptor 2 (VEGF‐R2) and CD68 (a marker for macrophages), indicating a positive effect on moderating hypoxia‐induced smooth muscle cell proliferation and inflammation without affecting the pulmonary levels of hypoxia‐inducible factor (HIF)‐1α. Furthermore, sildenafil depressed the number of CXCR4^+^ cells. Collectively, these findings indicate that the improvement in pulmonary haemodynamic by sildenafil is linked to decreased recruitment of BM‐derived c‐kit^+^ cells in the pulmonary tissue. The attenuation of the recruitment of BM‐derived c‐kit^+^ cells by sildenafil may provide novel therapeutic insights into the control of pulmonary remodelling.

## Introduction

Pulmonary hypertension (PH), a progressive disease of the pulmonary arterioles characterized by vascular remodelling that results in increased pulmonary arterial pressure 1, might lead to fatal right ventricular (RV) heart failure. Despite recent substantial progress in treatment, the prognosis remains poor 2, urging the need to identify new therapeutic targets. Chronic hypoxia (CH) contributes to PH 3 and is associated with many PH‐related diseases, including chronic obstructive pulmonary disease, cystic fibrosis, diffuse interstitial fibrosis, bronchopulmonary dysplasia, infiltrative lung tumours, congenital hearts defects and ischaemic events.

Previous reports show that the process of vascular remodelling induced by hypoxia involves the mobilization of bone marrow (BM)‐derived progenitor cells expressing the transmembrane tyrosine kinase receptor c‐kit 4, 5. BM‐derived c‐kit^+^ cells mobilization into the peripheral circulation creates an environment in the pulmonary artery vessel wall that facilitates adhesion of circulating cells. Many studies identified BM‐derived progenitors as important players in vascular pathology 4, 5, 6. Thus strategies that inhibit the recruitment of these cells to pulmonary arteries may constitute important therapeutic options for PH, and the tyrosine kinase inhibitors imatinib and sorafenib were found to alleviate PH in experimental models 6, 7. Although imatinib showed favourable effects in human beings 8, this drug has multiple targets with difficult prediction of the risk‐benefit ratio and may induce cardiotoxicity 9, 10, thus urging the need to explore alternative strategies. The effect of sildenafil, a non‐toxic phosphodiesterase‐5 (PDE‐5) inhibitor, on the loss of endothelium‐derived nitric oxide (NO) has provided a background for its use in the clinical treatment of PH 11, 12. By inhibiting cyclic guanosin mononucleotide (cGMP) breakdown to inactive 5′‐guanosin mononucleotide (5′‐GMP), sildenafil increases the phosphorylation and activation of the endothelial isoform of NOS (eNOS) and of protein kinase B (Akt), thereby reducing apoptosis 13, 14 and contrasting PH‐induced pathology. However, the underlying mechanism is not yet clear.

Rat breathing 10% O_2_ for 15 days develop RV hypertrophy that is partially corrected by sildenafil 15, 16. Furthermore, CH is a powerful activator of stromal cell‐derived factor‐1α (SDF‐1α) cell receptor, CXC chemokine receptor 4 (CXCR4) 6, 17, 18. This axis plays an important role in the migration of BM stem cells to lung lesions, as supported by data showing that its inhibition prevents c‐kit^+^ cell accumulation in pulmonary arteries and thus PH development 6, 17. Therefore, the aim of the present investigation was to test whether the mechanism underlying the attenuation of CH‐induced pulmonary remodelling by PDE‐5 inhibition might involve BM‐derived progenitor cells.

## Materials and Methods

### Animals and study design

Experiments were conducted on a total of 49 male eight‐week‐old Sprague Dawley rats (Charles River, France). Rats were fed standard diet without limitations until 24 hrs before killing. Room temperature was kept at 21 ± 2°C and 12 hrs of light was alternated with 12 hrs of dark. Experiments were performed in accordance with the Swiss federal law, the Guide for the Care and Use of Laboratory Animals published by the US National Institutes of Health (NIH Publication No. 85‐23, revised 1996) and the guidelines indicated in the Declaration of Helsinki of 1972. The protocol was approved by the Committee of the Ethics of Animal Experiments, Office Vétérinaire Cantonal, Lausanne. All efforts were made to minimize animal suffering.

Rats were subdivided into three groups: control (breathing atmospheric air at 21% O_2_; *n* = 10), CH rats (breathing air at 10% O_2_) treated with placebo (saline; *n* = 12) and CH rats treated with sildenafil (1.4 mg/kg/day, i.p., *n* = 12). All treatments had a fixed duration of 15 days. At the end of treatments, rats were anaerobically transferred into the compensation chamber flowed with either 21% or 10% O_2_, as described previously 15, 16, 19, 20 and anesthetized by i.p. injection of ketamine (100 mg/kg) plus xylazine (10 mg/kg).

### Haemodynamic measurements

The anesthetized rats were placed over a heating platform at 37°C and connected to a mechanical ventilator after tracheotomy (tidal volume 2.5 ml at 50 strokes/min.) using a Harvard Apparatus (model 683; Holliston, MA, USA) with either room air or hypoxic gas for control and hypoxic groups, respectively. To evaluate right ventricular (RV) pressure, the thorax was opened and a 25‐G needle was inserted into the RV apex, enabling the placement of a Millar Mikro‐Tip conductance catheter (model SPR‐838, tip size of 2F; Millar Instruments, Oxford, UK) along the long axis of the heart. After stabilization, the RV systolic pressure was recorded for each animal using an MPVS Ultra system (Millar Instruments). To evaluate the left ventricular (LV) pressure, the right carotid artery was cannulated with the Millar catheter, advanced into the LV cavity in the closed‐chest preparation and the LV systolic pressure was measured as described above.

### Blood gas analysis

Arterial blood was withdrawn from the left carotid artery of thoracotomized rats in a heparinized syringe and blood gasses were immediately measured (Servomex Oxygen Analyzer 570 A; Zurich, Switzerland). The base excess (BE) was calculated from arterial pH and PCO_2_ using the formula 21: BE = 0.02786 × PCO_2_ × 10^(pH ‐ 6.1)^ + 13.77 × pH ‐ 124.58. The remaining blood was centrifuged and plasma stored at ‐80°C for analysis of cGMP (ELISA kit; Assay designs, Inc., Ann Arbor, MI, USA).

### Lung morphology

At the end of the haemodynamic study, rats were killed with an overdose of anaesthesia, the lungs were inflated with 10% formalin at 25 cm H_2_O pressure through the trachea for 15 min., excised and finally processed for paraffin embedding. Lung sections (8 μm thick) were stained with an antibody against α‐smooth muscle actin (α‐SMA 1:250, clone 1A4; Sigma‐Aldrich, Buchs, Switzerland) overnight at 4°C, followed by a goat antimouse IgG secondary antibody (1:500, Dako, DK‐2600 Glostrup, Denmark), and all transversally cut arterioles were quantitatively analysed at 200× magnification using an images analysis system Nikon eclipse 80i camera and NIH image software (NIKON INSTRUMENTS INC. Melville, NY, USA).

Pulmonary arterial thickening was assessed by calculating the per cent pulmonary artery thickness using the following formula: 100 × (perivascular area‐luminal area)/luminal area. For this measurement, 20 arteries/sample of <50 μm were analysed. The degree of muscularization of small pulmonary arterioles was assessed from immunofluorescence staining with primary antibodies rabbit polyclonal α‐SMA (1:100, Abcam 5694) and mouse monoclonal PECAM‐1 (D‐11, 1:100; Santa Cruz, Dallas, TX, USA) and the secondary antibodies rabbit Alexa Fluor‐594 (1:500) or mouse Alexa Flour‐488 (1:500). Sections were examined using a confocal microscope. The percentage of muscularization of vessels from α‐SMA and PECAM‐1 florescence was analysed for threshold intensity and expressed as a percentage of total vessel area using imageJ software (NIH). All the morphological analysis was conducted in a double‐blind method.

### Immunofluorescence and immunohistochemical staining

The procedure for immunofluorescence staining is described elsewhere 22. The following antibodies (Santa Cruz) were used: c‐kit (H‐300 rabbit polyclonal, 1:100), VEGF‐R2 (A‐3, mouse monoclonal, 1:50) and CD68 (H‐255, rabbit polyclonal, 1:80). To detect colocalization with VEGF‐R2 or CD68, slides were double‐stained with mouse monoclonal VEGF‐R2 and mouse monoclonal CD68 and goat secondary antibody rhodamine‐conjugated (Santa Cruz), diluted 1:200 in PBS. Five distinct images per section were acquired by a Zeiss LSM 710 confocal microscope. Results are expressed as number of c‐kit^+^/VEGF‐R2^+^ or c‐kit^+^/CD68^+^ cells/vessel.

For CXCR4 and Ki‐67 immunofluorescence staining, antigen retrieval was performed by heat in citrate buffer (pH 6.0) for 20 min. Then, sections were washed and blocked for 1 hr at room temperature with 10% goat serum to saturate any non‐specific bindings, followed by overnight incubation at 4°C with the primary antibody CXCR4 (1:100, Santa Cruz) or Ki‐67 (ab66155). After washing, the sections were incubated with secondary antibody, Alexa 647‐conjugated goat anti‐rabbit IgG (1:400, Life Technologies Waltham, MA, USA) for 1 hr. All sections were counterstained with Hoechst 33342 staining (1:1000) and mounted with fluorescent mounting medium (Dako). Five sections per group were acquired using a Zeiss LSM 710 confocal microscope. Cell proliferation was quantified as number of Ki‐67‐positive cells/total nuclei and all CXCR4^+^ cells in the perivascular pulmonary arterioles were counted. Results are expressed as number of CXCR4^+^ cells/vessel. HIF‐1α immunohistochemical staining was performed as described 23.

### Biochemical measurements

An additional set of experiments (*n* = 15 rats) was performed for biochemical analysis. Lungs were quickly rinsed in ice‐cold PBS (pH 7.4) and clamped between steel tongs pre‐cooled with liquid N_2_ and stored at ‐80°C until analysis. For Western blotting analyses, frozen lung tissues were homogenized in lysis buffer plus protease inhibitor cocktail (Santa Cruz Biotechnology) and processed as described elsewhere 24, using the following primary antibodies: anti‐p‐eNOS (Ser^1177^, Cell Signalling Technology), anti‐eNOS (Santa Cruz Biotechnology), anti‐p‐Akt (Ser^473^) and anti‐Akt (both Cell Signaling Technologies). Band intensities were quantified using ImageJ software.

### Real‐time PCR analysis

Total RNAs of lung tissues were extracted using TRIzol reagent and reversed‐transcribed with oligo‐dT primers [Thermo Scientific (Waltham, MA, USA), Verso cDNA Synthesis Kit]. Quantitative RT‐PCR was performed with the use of QuantiStudio 6 Applied Biosystem real‐time detection system (Life technologies, Waltham, MA, USA) and the Fast SYBR Green Master Mix (Applied Biosystem, Waltham, MA, USA) following the manufacturer's instructions. PCR program was as follows: 94°C for 30 sec., 40 cycles of 95°C for 5 sec., 56°C for 30 sec., 72°C for 20 sec. The relative amount of mRNA expression for hypoxia‐inducible factor‐1α (HIF‐1α) was represented using the 2‐∆∆Ct value. The primer pairs for HIF‐1α PCR (138 bp) were (forward) 5′‐TGAGCTCACATCTTGATAAAGCTTCT‐3′ and (reverse) 5′‐GGGCTTTCAGATAAAAACAGTCCAT‐3′, and for the housekeeping gene GAPDH (359 bp) were (forward) 5′‐TGAAGGTCGGTGTGAACGGATTTG‐3′ and (reverse) 5′‐GGCGGAGATGATGACCCTTTTG‐3′, respectively.

### FACS analysis

Whole blood was mixed with 5 μM EDTA to prevent clotting, stained for 15 min. at room temperature with suitable combinations of the following monoclonal antibodies or isotype‐matched control monoclonal antibodies: CXCR4 FITC (Alomone Labs, Jerusalem, Israel) and CD177(c‐kit) PE (BD Pharmingen, Italy). Red blood cells were lysed with the use of BD Pharm Lyse (BD Biosciences, Milan) and analysed with the use of a Gallios Flow Cytometer (Beckman Coulter, Inc., Brea, CA, USA) equipped with Kaluza Software.

### Statistical analysis

Data are expressed as mean ± SEM. To detect differences among the groups, we used the one‐way anova test followed, if significant, by the Bonferroni's test to the differences between data pairs. Significance level was set at *P* = 0.05 (two‐tailed).

## Results

### Sildenafil restores cGMP levels and preserves survival pathways in hypoxia

**Figure 1:**
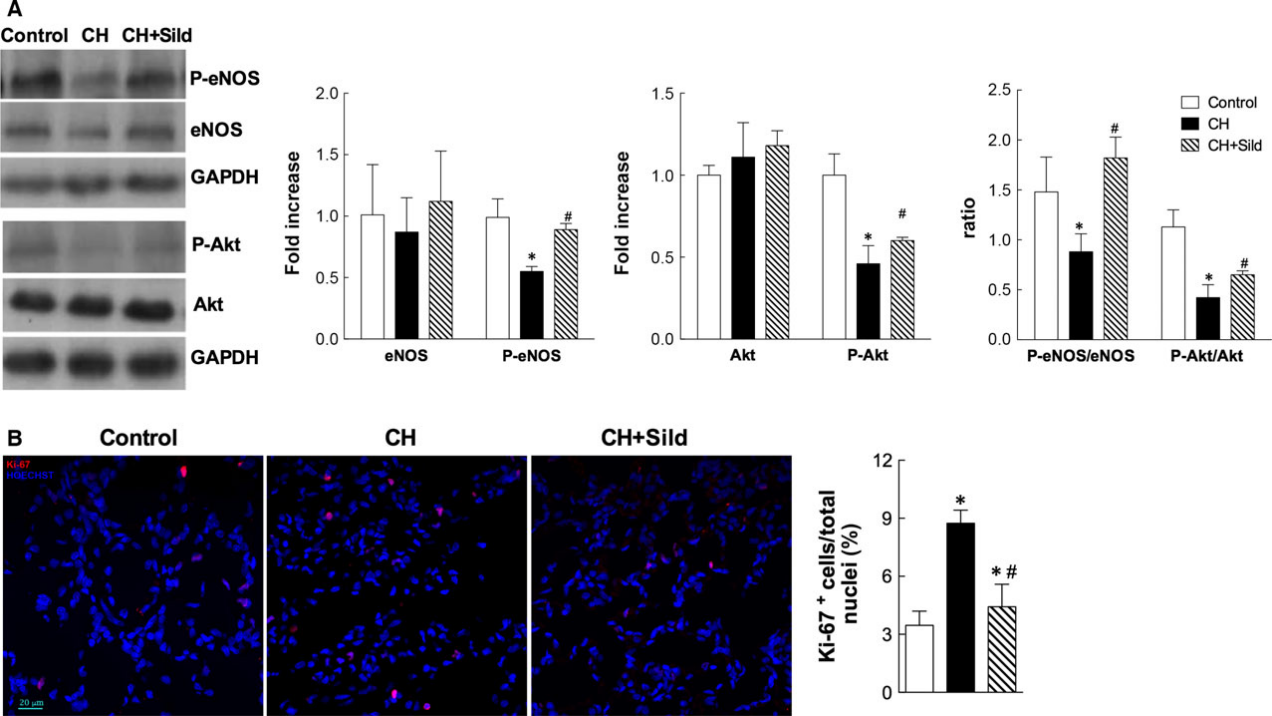
Sildenafil preserves survival pathways in hypoxia. (**A**) Left panel. Representative Western blots of whole pulmonary tissue homogenates for phospho‐eNOS (P‐eNOS), eNOS, phospho‐Akt (P‐Akt) and Akt. GAPDH was used as a loading control. Right panel. Densitometric analysis, calculated as fold increase versus control. (**B**) Left panel. Representative confocal immunofluorescence images of pulmonary tissue stained with antibody against Ki‐67 (red) with nuclei counterstained with HOECHST (blue). Right panel. Percentage of cells with a positive nuclear staining for Ki‐67 with respect to all nuclei. Scale bars = 20 μm. Data are presented as mean±SEM (n = 5/group); *P < 0.05 versus control, ^#^P < 0.05 versus chronic hypoxia (CH) (one‐way anova with Bonferroni post‐test).

Chronic hypoxia induced striking changes in blood gasses and base excess (Table 1), which were not affected by sildenafil. Plasma cGMP levels were higher in CH‐sildenafil rats compared with CH‐placebo and control rats, demonstrating that cGMP conversion to 5′‐GMP was efficiently inhibited by sildenafil. The expression of eNOS and Akt in pulmonary tissue did not change among groups (Fig. 1A), but phospho‐eNOS at Ser^1177^ and phospho‐Akt at Ser^473^ were decreased in CH compared with control and sildenafil was able to rescue such decrease. Additionally, sildenafil significantly increased both phospho‐eNOS/eNOS and phospho‐Akt/Akt ratios (Fig. 1A). As Akt is a key mediator of cell proliferation, we evaluated the level of proliferation in pulmonary tissue. CH increased Ki‐67‐positive nuclei, whereas sildenafil markedly reduced the degree of proliferation (Fig. 1B).

**Table 1:** Arterial blood gas analysis and blood values (mean ± SEM)

	Control	Chronic hypoxia + saline	Chronic hypoxia + sildenafil	anova *P*
*N*	15	17	17	
Weight gain, g	103 ± 5	13 ± 8*	3 ± 5*	<0.0001
PaO_2_, mmHg	83.5 ± 7.8	40.3 ± 2.8*	42.5 ± 2.8*	<0.0001
PaCO_2_, mmHg	39.3 ± 2.0	26.1 ± 2.5*	25.4 ± 1.3*	0.0001
pH	7.43 ± 0.01	7.40 ± 0.02	7.42 ± 0.02	NS
Base excess, mEq/l	0.7 ± 0.8	−8.3 ± 0.8*	−8.2 ± 0.5*	<0.0001
Haematocrit, %	41 ± 2	66 ± 1*	62 ± 1*, #	<0.0001
cGMP, nM	2.18 ± 0.32	2.34 ± 0.27	4.64 ± 0.91*, #	0.01

*P < 0.05 *versus* control, ^#^ *P* < 0.05 *versus* chronic hypoxia + saline (Bonferroni multiple comparison test).

### Sildenafil reduces hypoxia‐induced increase in c‐kit^+^ cell recruitment

**Figure 2:**
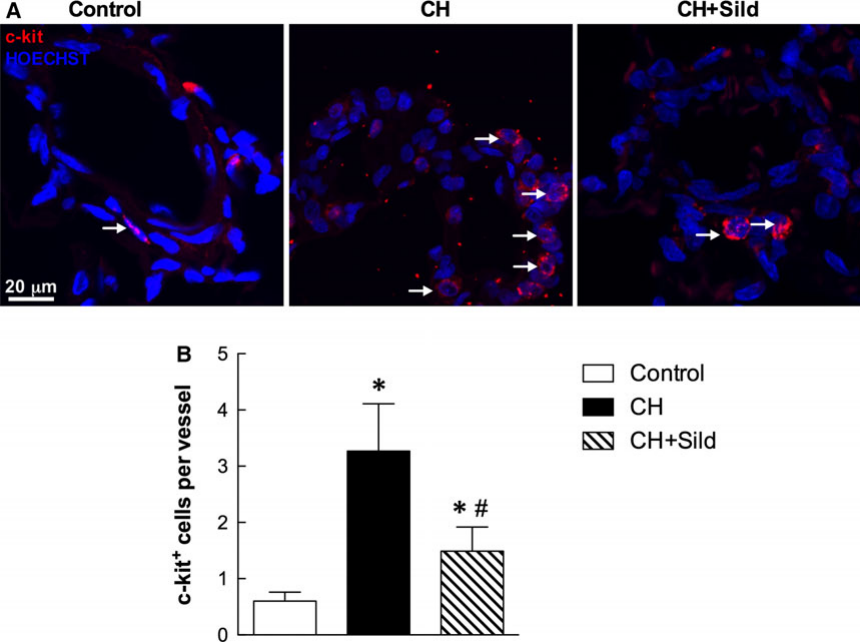
Sildenafil reduces hypoxia‐induced increase in c‐kit^+^ cell recruitment. (**A**) Representative confocal immunofluorescence staining for c‐kit^+^ cells (red). Nuclei were counterstained with HOECHST (blue). Scale bar = 20 μm. The plot (**B**) shows the quantification of the number of c‐kit^+^ cells/vessel (5 fields per section). Same details as in Fig. 1.

Emerging evidence suggests the importance of the BM‐derived progenitor cell population expressing the c‐kit marker in the process of vascular remodelling 25. Because sildenafil is able to modulate c‐kit^+^ cell mobilization into peripheral blood under CH (Fig. S1), we measured c‐kit^+^ cells mobilization in lungs. Immunostaining of the perivascular pulmonary artery showed that the number of c‐kit^+^ cells per vessel was higher in CH compared with control (Fig. 2). This effect was markedly reduced by sildenafil.

### Sildenafil modulates progenitor cell nature of c‐kit^+^ cells

**Figure 3:**
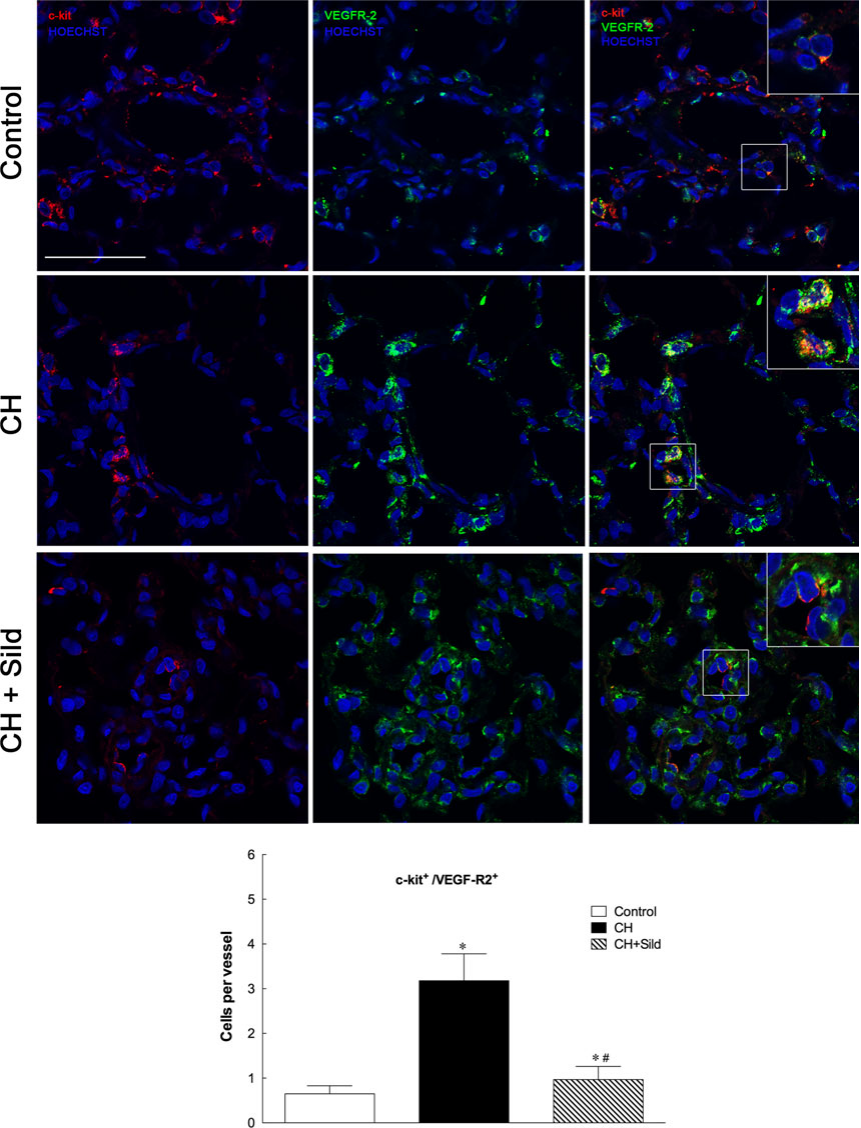
Sildenafil reduced hypoxia‐induced increase in c‐kit^+^/VEGF‐R2^+^g. Representative confocal immunofluorescence costainings for c‐kit^+^ (red) and VEGF‐R2 (green). Nuclei were counterstained with HOECHST (blue). Scale bars = 50 μm. The plot shows the quantification of the number of c‐kit^+^/VEGF‐R2^+^ cells (5 fields per section). Same details as in Fig. 1.

**Figure 4:**
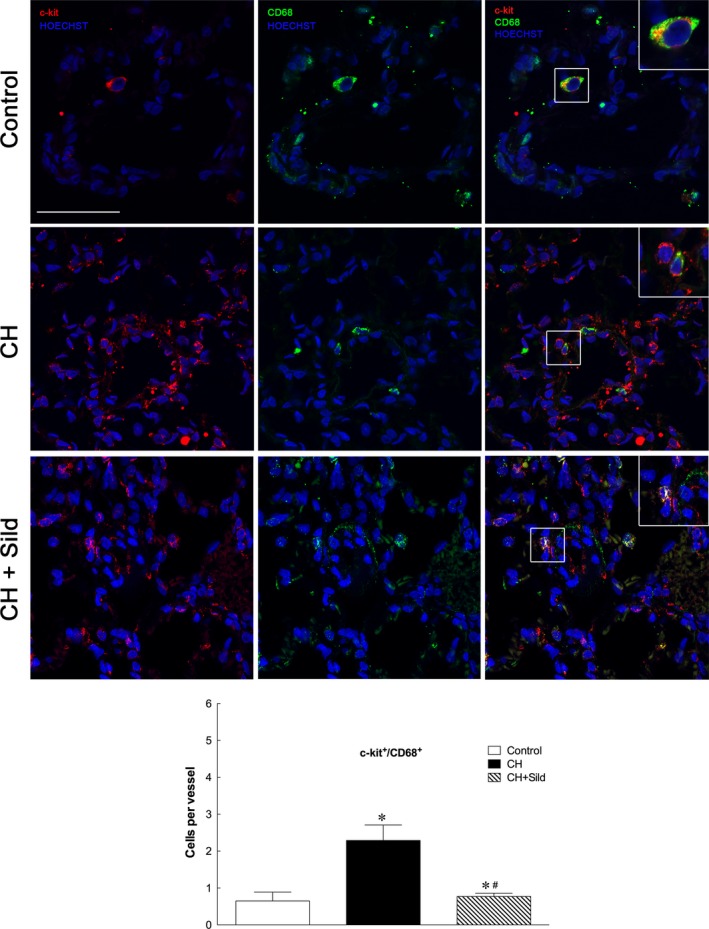
Sildenafil reduced hypoxia‐induced increase in c‐kit^+^/CD68^+^. Representative confocal immunofluorescence costainings for c‐kit^+^ (red) and CD‐68 (green). Nuclei were counterstained with HOECHST (blue). Scale bars = 50 μm. The plot shows the quantification of the number of c‐kit^+^/CD68 cells (5 fields per section). Same details as in Fig. 1.

**Figure 5:**
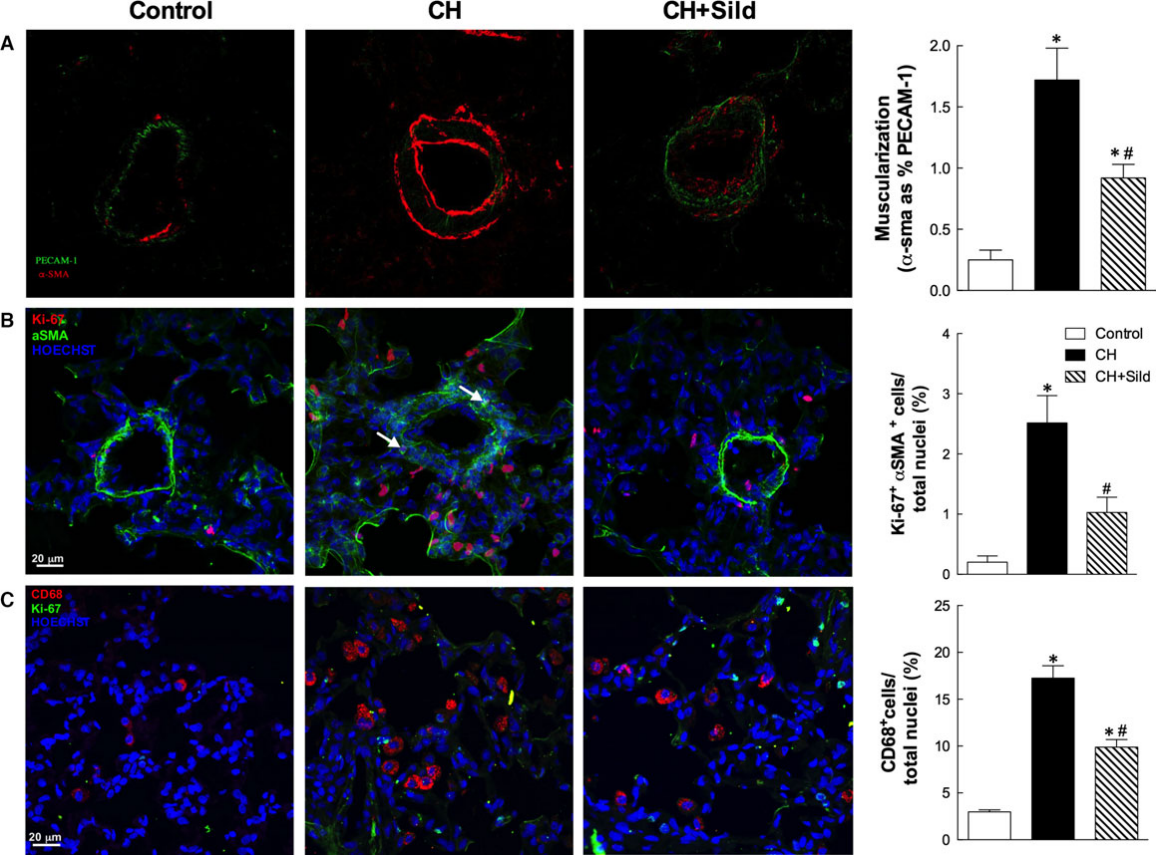
Effects of chronic hypoxia (CH) and sildenafil on muscularization and inflammation. (**A**) Left panel. Representative confocal images of vessels stained with α‐SMA (red) and PECAM‐1 (green). The plot (right panel) shows the index of muscularization of vessels calculated as per cent of α‐SMA staining on PECAM‐1‐positive vessels. Same details as in Fig. 1. (**B**) Representative confocal immunofluorescence staining of pulmonary sections for α‐SMA (green) and Ki‐67 (red). Nuclei were counterstained with HOECHST (blue). Scale bars = 20 μm. (**C**) Representative confocal immunofluorescence staining of pulmonary sections for CD68 (red) and Ki‐67 (green). Nuclei were counterstained with HOECHST (blue). Scale bars = 20 μm.

Chronic hypoxia significantly increased the number of c‐kit^+^/VEGF‐R2^+^ (Fig. 3) and c‐kit^+^/CD68^+^ cells (Fig. 4) compared with control and sildenafil reduced this number. This indicates important effects of CH and sildenafil on muscularization and inflammation. To assess the first, we measured the level of muscularized *versus* non‐muscularized vessels by α‐SMA and PECAM‐1 costaining as described in Materials and Methods. Figure 5A shows that whereas CH increased muscularization, sildenafil reversed remodelling. Consistently, in three representative confocal immunofluorescence images targeted at identifying vascular smooth muscle cells (VSMC), we found that sildenafil reduced VSMC proliferation (Fig. 5B). To support the occurrence of inflammatory processes, we measured by confocal microscopy the recruitment of CD68 cells and found that sildenafil could reduce such recruitment (Fig. 5C).

### Sildenafil regulates mobilization and recruitment of CXCR4^+^ cells

**Figure 6:**
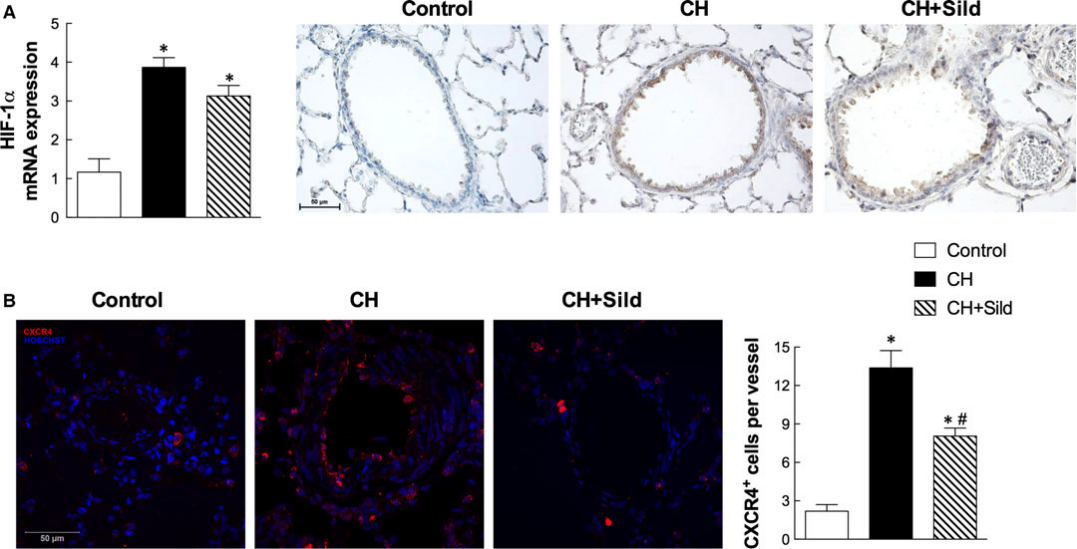
Sildenafil regulates mobilization and recruitment of CXCR4^+^ cells. (**A**) Left panel. mRNA quantification of HIF‐1α. Same details as in Fig. 1. Right panel. Immunohistochemical staining (brown) of HIF‐1α in haematoxylin pulmonary sections. (**B**) Left panel. Representative confocal microscopy images for CXCR4 (red) in pulmonary tissue. Nuclei were counterstained with HOECHST (blue). Scale bar = 50 μm. The right panel shows the number of CXCR4^+^ cells per vessel (5 fields per section). Same details as in Fig. 1.

Chronic hypoxia increased mRNA expression of the hypoxia‐inducible factor (HIF)‐1α (Fig. 6A left panel). However, sildenafil did not change it, supporting the view that sildenafil did not act on the degree of hypoxia (see also Table 1). Furthermore, immunohistochemical staining showed that HIF‐1α expression was higher in CH than in control pulmonary tissue (Fig. 6A, right panel) and was not affected by sildenafil. Because CH increased the plasma level of CXCR4^+^ cells and sildenafil tended to blunt such increase (Fig. S2), we evaluated the mobilization and recruitment of CXCR4^+^ cells in pulmonary tissue by confocal immunostaining. Whereas CH increased the number of CXCR4^+^ cells in the perivascular wall of small pulmonary arteries (Fig. 6B), sildenafil attenuated the number of these cells, further supporting the antimobilization role of sildenafil.

### Outcome of altered recruitment of c‐kit^+^ cells

**Figure 7:**
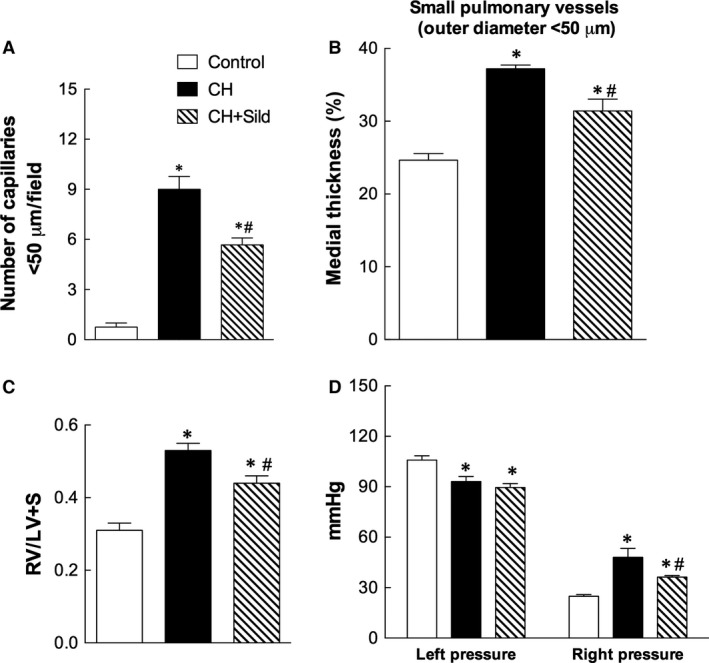
Outcome of altered recruitment of c‐kit^+^ cells. (**A**) Quantification in 10 fields/sample of the number of vessels with diameter <50 μm. (**B**) Morphometric analysis performed on 20 pulmonary vessels (<50 μm diameter) to assess the percentage of medial wall thickness of pulmonary arteries. (**C**) Right ventricular hypertrophy, calculated as the weight of RV/left ventricle + septum. Right panel. (**D**) Left and the right ventricular pressures. Same details as in Fig. 1.

To understand how the phenomena described above translate into clinically relevant outcome, we explored angiogenesis and some downstream physiological effects. We first calculated the number of lung tissue vessels <50 μm diameter by α‐SMA staining (Fig. 7A). CH increased this value and sildenafil attenuated such increase. We also assessed the degree of pulmonary vascular remodelling by measuring the medial wall thickness of small pulmonary arteries (Fig. 7B). Compared with control, CH group had a significant increase in medial wall thickness, which was attenuated by sildenafil treatment. To assess the cardiopulmonary effect of sildenafil during CH, we measured the right ventricle hypertrophy and the cardiac load in the LV and RV (Fig. 7C and D, respectively). While CH induced an increase in the RV/LV + S weight ratio, sildenafil nearly blunted this increase. Furthermore, whereas LV pressures were only marginally affected, RV pressures were increased in CH compared with control rats. Treatment with sildenafil decreased RV systolic pressure, indicating that it may attenuate the development of CH‐induced PH.

## Discussion

Data gathered in this study support the hypothesis that sildenafil, a PDE‐5 inhibitor, attenuates CH‐induced pulmonary vascular remodelling and dysfunction by modulating BM‐derived c‐kit^+^ progenitor cell recruitment into the lungs. This finding is particularly relevant in the context of PH because it was recently reported that BM‐derived cells are the key drivers of lung remodelling and inflammation in a mouse model of this condition 26. On this basis, it can be argued that reducing mobilization/recruitment of BM cells during CH may represent a good strategy to prevent adverse pulmonary remodelling.

Here, to further shed light on these processes and in the attempt to find novel mechanistic insights, we employed a well‐established CH model 19 to establish the effect of sildenafil (1.4 mg/kg i.p., a dose compatible with that used for treatment of heart failure in human beings 14) in PH.

As expected 4, 5, 6, CH enhanced the number of c‐kit^+^ cells, a specific population of BM cells, in pulmonary artery (Fig. 2) and was associated with increased small pulmonary vessels (Fig. 7A). This is in agreement with previous investigation showing that c‐kit^+^ cells participate in the new vessel formation process during chronic hypoxia 5. To substantiate these findings, we showed that CH markedly increases the number of c‐kit^+^ cells colocalizing with VEGF‐R2 and CD68 receptors. As VEGF‐2R is a marker for pro‐angiogenesis progenitor cells 27, the accumulation of c‐kit^+^/VEGF‐R2^+^ cells in CH might imply greater potential for the genesis of pulmonary arteries. We found that CH augments the number of c‐kit^+^/CD68^+^ cells (monocytes/macrophages), consistently with a previous report showing increased infiltration of inflammatory cells in pulmonary tissues of patients with idiopathic PH 28.

This study provides the first evidence that sildenafil prevents small pulmonary vessel remodelling in CH contrasting the recruitment of all the cell types mentioned above that express the c‐kit marker in the hypoxic lung. Although not conclusive, the presented data suggest that sildenafil reduces the potential deleterious effects of c‐kit^+^ cells in the CH scenario. Indeed, a pathogenic role for c‐kit^+^ cells in CH was recently suggested by the finding that imatinib, a tyrosine kinase inhibitor blocking c‐kit receptor, improves pulmonary arterial remodelling by decreasing the recruitment of c‐kit^+^ cells in pulmonary arterial lesions of hypoxic mice 6.

However, we cannot exclude that sildenafil prevents reverse remodelling in lung during CH by acting also on other cell types and with parallel mechanisms. In facts, we and others 29 have shown that sildenafil attenuates at least two mechanisms that are crucial in the process of vascular remodelling: the hypoxia‐induced VSMC proliferation and the hypoxia‐induced inflammatory cell recruitment. Whereas VSMC phenotypic switching contributes to the structural changes observed in the pulmonary arteries during CH 30, emerging evidence demonstrates that inflammatory cells accumulate at perivascular sites in the hypoxic lungs 30.

Noteworthy, sildenafil also attenuates CXCR4^+^ mobilization and the subsequent recruitment into the remodelled pulmonary arteries (Fig. 6B). The chemokine receptor CXCR4 is highly expressed in immune cells and mediates the migration of resting leucocytes and haematopoietic progenitors in response to its ligand, SDF‐1α. The latter and its receptor CXCR4 have been shown to be critical for homing and mobilization of c‐kit^+^ progenitor cells in response to tissue hypoxia 31, 32. Thus, we believe that sildenafil decreases c‐kit^+^ cell recruitment to the damaged lung mainly by acting on the SDF‐1α/CXCR4 axis. To further support this paradigm, it was reported that the administration of AMD3100, a CXCR4 antagonist, attenuates hypoxia‐induced PH by reducing pulmonary c‐kit^+^ progenitor cell accumulation 6.

Intriguingly, in this study sildenafil reduced the number of CXCR4^+^ progenitor cells in the pulmonary artery adventitia but not the CH‐induced expression of HIF‐1α. This indicates that sildenafil decreases CXCR4^+^ cells recruitment without affecting the degree of hypoxia, as confirmed by conserved haematocrit and blood gas analysis (Table 1). It is thus likely that the beneficial effects of sildenafil remain linked, at least in part, to the activation of the NO/cGMP and the PI3K/Akt signalling pathways. Remarkably, the recovery in Akt/eNOS activation by sildenafil suggests a mechanism with relevant impact on the inflammatory response. Indeed, it was reported that Akt is upstream eNOS phosphorylation 33 and that eNOS‐derived NO plays an important role in inhibiting macrophage infiltration into the lungs of PH patients 34.

In conclusion, this study provides the first evidence that PDE‐5 inhibition by sildenafil is associated with a reduction in the mobilization and homing of c‐kit^+^ cells to the hypoxic lung. This association may be linked to the effect of sildenafil in the prevention of pulmonary vascular remodelling induced by CH. The underlying mechanism appears to involve the modulation of the SDF‐1α/CXCR4 axis as well as the Akt/eNOS pathway to contrast the inflammatory pulmonary drive during hypoxia. These results highlight a novel paradigm for the clinical use of sildenafil in the management of PH.

## Conflicts of interest

The authors confirm that there are no conflicts of interest.

## Supporting information


**Figure S1** Representative histogram plot for c‐kit marker in whole blood measured by FACS (left panel); right panel indicates the quantification of circulating c‐kit^+^ cells. Same details as in the manuscript.
**Figure S2** FACS analysis for CXCR4 marker in WB cells: left panel shows a representative histogram plot, the right panel indicates the quantification of circulating CXCR4^+^ cells. Same details as in the manuscript.
