# Supplementary material for: Sildenafil attenuates hypoxic pulmonary remodelling by inhibiting bone marrow progenitor cells
Source: J Cell Mol Med. 2016 Nov 18;21(5):871–80. doi: 10.1111/jcmm.13026 (PMC5387166; doi:10.1111/jcmm.13026)
Supplement: Supplementary file 1 — Figure S1 Representative histogram plot for c‐kit marker in whole blood measured by FACS (left panel); right panel indicates the quantification of circulating c‐kit+ cells. Same details as in the manuscript. Figure S2 FACS analysis for CXCR4 marker in WB cells: left panel shows a representative histogram plot, the right panel indicates the quantification of circulating CXCR4+ cells. Same details as in the manuscript. [file JCMM-21-871-s001.docx]

Sildenafil attenuates hypoxic pulmonary remodeling by inhibiting bone marrow progenitor cells

SUPPLEMENTAL DATA

Shirley Favre^1^, Elisa Gambini^2^, Patrizia Nigro^2^, Alessandro Scopece^2^, Paola Bianciardi^3^, Anna Caretti^3^, Giulio Pompilio^2^, Antonio F Corno^4^, Giuseppe Vassalli^5^, Ludwig K von Segesser^1^, Michele Samaja^3^, and Giuseppina Milano^1,2*^

^1^Laboratory of Cardiovascular Research, Department of Surgery and Anesthesiology, University Hospital Lausanne, Lausanne, Switzerland; ^2^Vascular Biology and Regenerative Medicine Unit, Centro Cardiologico Monzino–IRCCS, Milan, Italy; ^3^Department of Health Science, University of Milan, Milan, Italy; ^4^Glenfield Hospital, Leicester, United Kingdom; ^5^Laboratory of Molecular and Cellular Cardiology, Departments of Cardiology and Heart Surgery, Lausanne, Switzerland.

*****Corresponding Author: Laboratory of Cardiovascular Research, Department of Surgery and Anaesthesiology, University Hospital Lausanne, rue du Bugnon 46, 1011 Lausanne, Switzerland, Tel.: ++41 21 3142297, e-mail: [giuseppina.milano@chuv.ch](mailto:giuseppina.milano@chuv.ch)

**Running title:** Sildenafil and bone marrow progenitor in lung

**Supplementary Figure 1S.**

Representative histogram plot for c-kit marker in whole blood measured by FACS (left panel); right panel indicates the quantification of circulating c-kit^+^ cells. Same details as in the manuscript.

**Supplementary Figure 2S.** FACS analysis for CXCR4 marker in WB cells: left panel shows a representative histogram plot, the right panel indicates the quantification of circulating CXCR4^+^ cells. Same details as in the manuscript.
